# Eating habits and lifestyle changes during COVID-19 lockdown: an Italian survey

**DOI:** 10.1186/s12967-020-02399-5

**Published:** 2020-06-08

**Authors:** Laura Di Renzo, Paola Gualtieri, Francesca Pivari, Laura Soldati, Alda Attinà, Giulia Cinelli, Claudia Leggeri, Giovanna Caparello, Luigi Barrea, Francesco Scerbo, Ernesto Esposito, Antonino De Lorenzo

**Affiliations:** 1 Section of Clinical Nutrition and Nutrigenomic, Department of Biomedicine and Prevention, University of Tor Vergata, Via Montpellier 1, 00133 Rome, Italy; 2 Department of Health Sciences, University of Milan, Via A. Di Rudinì, 8, 20142 Milan, Italy; 3 School of Specialization in Food Sciences, University of Rome Tor Vergata, Via Montpellier 1, 00133 Rome, Italy; 4 Predictive and Preventive Medicine Research Unit, “Bambino Gesù” Children Hospital IRCCS, 00165 Rome, Italy; 5 Dipartimento di Medicina Clinica e Chirurgia, Unit of Endocrinology, Federico II University Medical School of Naples, 80131 Naples, Italy; 6 Doctoral School in Public Health and Nursing, “Policlinico Tor Vergata” Foundation, Rome, Italy; 7 General Directorate for the Department of Human Policies, Basilicata Region, Italy

**Keywords:** COVID-19, Coronavirus, Mediterranean diet, Eating habits, Lifestyle

## Abstract

**Background:**

On December 12th 2019, a new coronavirus (SARS-Cov2) emerged in Wuhan, China, sparking a pandemic of acute respiratory syndrome in humans (COVID-19). On the 24th of April 2020, the number of COVID-19 deaths in the world, according to the COVID-Case Tracker by Johns Hopkins University, was 195,313, and the number of COVID-19 confirmed cases was 2,783,512. The COVID-19 pandemic represents a massive impact on human health, causing sudden lifestyle changes, through social distancing and isolation at home, with social and economic consequences. Optimizing public health during this pandemic requires not only knowledge from the medical and biological sciences, but also of all human sciences related to lifestyle, social and behavioural studies, including dietary habits and lifestyle.

**Methods:**

Our study aimed to investigate the immediate impact of the COVID-19 pandemic on eating habits and lifestyle changes among the Italian population aged ≥ 12 years. The study comprised a structured questionnaire packet that inquired demographic information (age, gender, place of residence, current employment); anthropometric data (reported weight and height); dietary habits information (adherence to the Mediterranean diet, daily intake of certain foods, food frequency, and number of meals/day); lifestyle habits information (grocery shopping, habit of smoking, sleep quality and physical activity). The survey was conducted from the 5th to the 24th of April 2020.

**Results:**

A total of 3533 respondents have been included in the study, aged between 12 and 86 years (76.1% females). The perception of weight gain was observed in 48.6% of the population; 3.3% of smokers decided to quit smoking; a slight increased physical activity has been reported, especially for bodyweight training, in 38.3% of respondents; the population group aged 18–30 years resulted in having a higher adherence to the Mediterranean diet when compared to the younger and the elderly population (p < 0.001; p < 0.001, respectively); 15% of respondents turned to farmers or organic, purchasing fruits and vegetables, especially in the North and Center of Italy, where BMI values were lower.

**Conclusions:**

In this study, we have provided for the first time data on the Italian population lifestyle, eating habits and adherence to the Mediterranean Diet pattern during the COVID-19 lockdown. However, as the COVID-19 pandemic is ongoing, our data need to be confirmed and investigated in future more extensive population studies.

## Background

The 2019 Coronavirus Disease or, as it is now called, COVID-19, is a severe acute respiratory syndrome caused by SARS coronavirus 2 (SARS-CoV-2). It was supposed that in December 2019, SARS-CoV-2 apparently transit from animals to humans at the Huanan seafood market and rapidly spread from Wuhan City of Hubei, Province of China, to the rest of the world [1]. Due to the growing case notification rates at Chinese and international locations, on the 30th January 2020, the WHO Emergency Committee declared a global health emergency [2]. In order to contrast and contain the spread of the new COVID-19, at the beginning of March 2020, the Italian Government decided for more stringent containment measures: the ban on mass gatherings and events, as well as the ban on meeting up for no urgent reasons, were issued on the entire national territory [3]. In particular, after almost a month of lockdown, as of March 28th, 2020, Italy with 92.472 cases and 10.023, was the second world’s worst-affected country in the COVID-19 pandemic [4]. In detail, the percentage of the new positive cases in Italy showed an average growth rate of +19.63%, with the highest percentage the on February 27th of +52.73% and a lowest percentage on March 28th of +5.50% [4]. Due to the *#iorestoacasa* decree [5] (translated as #stayathome decree), a sudden and radical change has occurred in the habits and lifestyles of the population, with a drastic reduction of any form of socialisation. Physical distancing and self-isolation strongly impacted citizens’ lives, affecting in particular eating habits and everyday behaviours.

There are two major influences: staying at home (which includes digital-education, smart working, limitation of outdoors and in-gym physical activity) and stockpiling food, due to the restriction in grocery shopping. In addition, the interruption of the work routine caused by the quarantine could result in boredom, which in turn is associated with a greater energy intake [6]. In addition to boredom, hearing or reading continuously about the COVID-19 from media can be stressful. Stress leads subjects toward overeating, especially ‘comfort foods’ rich in sugar, defined as “food craving” [7, 8]. Those foods, mainly rich in simple carbohydrates, can reduce stress as they encourage serotonin production with a positive effect on mood [9]. However, this food craving effect of carbohydrates is proportional to the glycemic index of foods that is associated with the increased risk of developing obesity and cardiovascular diseases, beyond a chronic state of inflammation, that has been demonstrated to increase the risk for more severe complications of COVID-19 [10, 11].

This new condition may compromise maintaining a healthy and varied diet, as well as a regular physical activity. For example, limited access to daily grocery shopping may lead to reduce the consumption of fresh foods, especially fruit, vegetables and fish, in favour of highly processed ones, such as convenience foods, junk foods, snacks, and ready-to-eat cereals, which tend to be high in fats, sugars, and salt. Moreover, psychological and emotional responses to the COVID-19 outbreak [12, 13], may increase the risk of developing dysfunctional eating behaviors. It is well known how the experience of negative emotions can lead to overeating, the so-called “emotional eating” [14, 15]. In order to contrast and respond to the negative experience of self-isolation, people could be more prone to look for reward and gratification physiologically associated with food consumption, even overriding other signals of satiety and hunger [16]. In addition, boredom feelings, which may arise from staying home for an extended period, are often related to overeating as a means to escape monotony [17, 18]. On the other hand, negative experiences may lead to eating restriction, due to the physiological stress reactions that mimic the internal sensations associated with feeding-induced satiety.

Finally, lifestyle may be substantially changed due to the containment measures, with the consequent risk of sedentary behaviours, modification in smoking and sleeping habits. Of interest, different studies reported an association between sleep disturbances and obesity due to increase the secretion of pro-inflammatory cytokines by the increased visceral adipose that could contribute to alter the sleep–wake rhythm [19, 20]. In addition, also diet seems to influence the quality of sleep, in fact very recently in a cross-sectional study included 172 middle-aged adults it has been reported that good sleepers had higher adherence to the Mediterranean diet (MD) and lower body mass index (BMI) compared to poor sleepers [21].

Considering the smoking, there are a significant association exists between SARS-CoV-2 infection and air pollution, and in this context in smokers, more severe COVID-19 symptoms occur [22].

Low physical activity levels have been suggested to interact both with body fat and appetite dysregulation [23].

Eating habits and lifestyle modification may threaten our health. Maintaining a correct nutrition status is crucial, especially in a period when the immune system might need to fight back. In fact, subjects with severe obesity (BMI ≥ 40 kg/m^2^) are one of the groups with the higher risk for COVID-19 complications [24]. Obesity is an expansion of the adipose tissue, which produces cytokines and contributes to a proinflammatory milieu [25]. Moreover, in regards to pulmonary physiology, subjects with obesity have decreased expiratory reserve volume, functional capacity and respiratory system compliance. In patients with high abdominal fat, pulmonary function is further compromised in the supine position by decreased diaphragmatic excursion, making ventilation more difficult [26]. The inflammatory state is also one of the most important factors of the severity of lung disease in COVID-19, which leads to the famous “*cytokine storm”* associated with the acute respiratory distress syndrome and multiple organ failure [11]. In this complex *scenario,* the inflammatory state characteristics in individuals with obesity could further exacerbate the inflammation in patients with COVID-19, therefore, exposing them to a higher concentration of proinflammatory cytokines compared to normal-weight individuals [11].

Further, following a healthy diet is important because gene expression levels of all the cytokines are influenced by food [27] and are capable of modulating the processes of inflammation and oxidative stress [27]. Several studies have confirmed an inverse association between the adherence to the MD and the overall cancer-related mortality. The healthy MD [28] is a proper combination of quality foods, based on macro and micronutrient content, and the absence of contaminating substances. According to current knowledge, the MD is the key factor against immune-mediated inflammatory responses, such as those occurring in cancer. In particular, their potential clinical applications are, on one side, low cholesterol levels and, on the other hand, high levels of antioxidants contained in fruits and vegetables, and monounsaturated fatty acid (MUFA), present in fish, nuts and olive oil [29]. Notably, it is well known that the MD, one of the healthiest dietetic pattern in the world, is linked to lower mortality and reduction in obesity, type 2 diabetes mellitus, low-grade inflammation, cancer, Alzheimer’s disease, depression, and Crohn’s disease [29, 30].

In light of the above, the “Eating Habits and Lifestyle Changes in COVID19 lockdown” (EHLC-COVID19) project began by using a web-survey. The main aim of the project, from a diachronic perspective, is to explore and analyse the changes in eating behavior and adherence to the MD and lifestyle during lockdown among the Italian population, according to the regional distribution of the COVID-19 epidemic and to age. Secondly, it allows to achieve nutritional interventions in supporting the health status of different target groups of the population, according to geographical distribution.

## Materials and methods

### Survey methodology

The EHLC-COVID19 project was carried out by the Section of Clinical Nutrition and Nutrigenomic, Department of Biomedicine and Prevention of the University of Rome Tor Vergata, using a web-survey to obtain data, from every Italian region, about people eating habits and lifestyle during the COVID-19 pandemic.

The survey was conducted from the 5th to the 24th of April 2020, among the Italian population, by using an online platform, accessible through any device with an Internet connection. The survey was disseminated through institutional and private social networks (Twitter, Facebook, and Instagram), the “*PATTO in Cucina* Magazine” website [31], and institutional mailing lists. This method of administration provides a statistical collective whose population parameters cannot be controlled as it is the case for probabilistic sampling. However, it was completely effective for the research objectives, because it facilitated the wide dissemination of the survey questionnaire during a period where, due to the pandemic, there are many territorial restrictions. Moreover, the latest data reported by the annual Italian report on the use of the internet shows that Internet penetration stood at 82% in January 2020; in particular, 94% of internet users, aged 16 to 64, use their smartphone to connect and 99% of them visited or used a social network or messaging services [32].

### EHLC-COVID19 questionnaire

The EHLC-COVID19 questionnaire was specifically built by using Google Form by the Section of Clinical Nutrition and Nutrigenomic, Department of Biomedicine and Prevention of the University of Rome Tor Vergata. The questionnaire, included 43 questions divided into four different sections: (1) personal data (4 questions: age, gender, hometown, current employment—especially if they had the possibility to work from home, also called “smart working”); (2) anthropometrics information (2 questions: reported weight and height); (3) dietary habits information: (a) adherence to the MD, using the validated 14-items Mediterranean diet adherence screener (MEDAS), which score ranges from 0 to 14 points [33], (b) structured questionnaire packet (11 questions: daily consumption of certain foods—for example junk food consumption: packaged sweets and baked products, sweet beverages, salted snacks and dressing sauces; food frequency; number of meals/day); (4) lifestyle habits information (12 questions: grocery shopping, smoke habit, hours of sleep and physical activity). Specific questions about physical activity habits were modified from a survey conducted by Istituto Superiore di Sanità [34]. The full version of the questionnaire is available as Appendix. The score of the adherence to the MD was assessed using MEDAS questionnaire [33]. On the basis of the MEDAS values, participants were divided in three classes: (1) low adherence (score 0–5), (2) medium adherence (score 6–9) and (3) high (score ≥ 10) adherence to the MD and differences in the compliance rates for each food were calculated.

The study was conducted in full agreement with the national and international regulations, and the Declaration of Helsinki (2000). All participants were fully informed about the study requirements and were required to accept the data sharing and privacy policy before participating in the study. Participants completed the questionnaire directly connected to the Google platform. Participants’ personal information, including names, were anonymized to maintain and protect confidentiality. The anonymous nature of the web-survey does not allow to trace in any way sensitive personal data. Therefore, the present web-survey study does not require approval by Ethics Committee. Once completed, each questionnaire was transmitted to the Google platform and the final database was downloaded as a Microsoft Excel sheet.

### Statistical analyses

Data are represented as number and percentage in parentheses (%) for categorical variables, or median and interquartile range in square brackets [IQR] for continuous variables. The Shapiro–Wilk test was performed in order to evaluate variables distribution. All the variables had skewed distribution. The Spearman correlation coefficient was calculated in order to evaluate the correlation between continuous variables. Chi square test was employed to assess the association between categorical variables while McNeman analysis was used to investigate the difference between categorical variables pre and during the COVID-19 emergency. Instead, Mann–Whitney U and Kruskal–Wallis tests were performed to compare continuous variables among two or more groups, respectively. Finally, binary and multinomial logistic regression analyses were conducted to investigate the association between categorical variables (dependent) and continuous or categorical ones (independent). Results were significant for *p* value < 0.05. Statistical analysis was performed using SPSS ver. 21.0 (IBM, Chicago, IL, USA).

## Results

### Participants

On the 24th of April 2020, the web-survey was concluded, and the collected data were analysed. A total of 4500 participants completed the questionnaire, and, after validation of the data, 3533 respondents have been included in the study, aged between 12 and 86 years. The female respondents represent 76.1% of the population.

According to gender and age distribution, the sample reflects the population of Italian Internet users (i.e., 91.4% of people older than 20 years) [35]. Territorial coverage spreads over all the Italian Regions: 15.5% of respondents live in Northern Italy, 56.9% in Center Italy, and 27.6% in Southern Italy and Islands. General characteristics and anthropometrics of the population are reported in Table 1. The Kruskal–Wallis test showed a statistically significant difference in BMI among the three Italian areas, and, in particular in the post hoc analysis, South and Islands resulted in having a population with higher BMI when compared to North and Center Italy (p = 0.007, p = 0.008; respectively). In terms of employment status, 1042 (29.5%) participants have a full-time job in smart working, 429 (12.1%) go to the workplace, 674 (19.1%) are students, 289 (8.2%) are unemployed, 940 (26.6%) suspended work and 159 (4.5%) are retired.

**Table 1:** Participants’ general characteristics and anthropometrics

	Whole sample (n = 3533)	Northern Italy (n = 547)	Center Italy (n = 2009)	Southern Italy and Islands (n = 977)
Age (years)	36.0 [27.0–49.0]* 40.03 ± 13.53	36.0 [29.0–52.0] 40.10 ± 13.48	37.0 [27.0–50.5] 39.29 ± 14.37	35.0 [26.0–45.0] 35.97 ± 13.87
Age groups (years)				
< 18	180 (5.1)	2 (0.4)	67 (3.3)	111 (11.4)
18–30	1048 (29.7)	177 (32.4)	616 (30.7)	255 (26.1)
31–50	1492 (42.2)	217 (39.7)	824 (41.0)	451 (46.2)
51–65	693 (19.6)	135 (24.7)	420 (20.9)	138 (14.1)
> 65	120 (3.4)	16 (2.9)	82 (4.1)	22 (2.3)
Gender (F)	2689 (76.1)	435 (79.5)	1313 (76.4)	738 (75.5)
Weight (kg)	65.0 [57.0–75.0]* 66.87 ± 13.16	65.0 [57.0–75.0] 66.88 ± 13.15	65.0 [57.0–75.0] 67.27 ± 14.35	65.0 [56.8–76.0] 67.74 ± 14.42
Height (cm)	166.0 [160.0–172.0]* 167.58 ± 8.47	167.0 [162.0–173.0] 167.60 ± 8.47	167.0 [161.0–173.0] 167.43 ± 8.25	165.0 [160.0–170.0] 166.37 ± 8.10
BMI (kg/m^2^)	23.23 [21.01–26.01]* 27.66 ± 4.10	22.89 [20.83–25.47] 23.76 ± 4.10	23.15 [20.94–25.87] 23.89 ± 4.29	23.53 [21.26–26.57] 24.37 ± 4.30
Class of BMI				
Underweight	142 (4.0)	19 (3.5)	97 (4.8)	26 (2.7)
Normal weight	2243 (63.5)	368 (67.3)	1273 (63.4)	602 (61.6)
Overweight	814 (23.0	108 (19.7)	469 (23.3)	237 (24.3)
Obesity I	251 (7.1)	42 (7.7)	1 26 (6.3)	83 (8.5)
Obesity II	66 (1.9)	8 (1.5)	32 (1.6)	26 (2.7)
Obesity III	17 (0.5)	2 (0.4)	12 (0.6)	3 (0.3)

Values are expressed as median and IQR in square brackets (M [IQR]) as well as mean and standard deviation (M ± SD) for continuous variables or as number and percentage (n (%)) for categorical variables

*BMI* body mass index

*The Shapiro–Wilk test was performed to evaluate variables distribution. Variables are considered non-normally distributed for p < 0.05

### Lifestyle changes during COVID-19 emergency

With regards to lifestyle changes during the COVID-19 lockdown, most of the population declares not to have changed its habits (46.1%), while 16.7% and 37.2% feel to have improved them or made them worse, respectively. In particular, smoking habits have been reduced during the lockdown (McNemar value = 101.484, p < 0.001), and sleep hours have increased (McNemar value = 330.851, p < 0.001), as shown in Table 2, equally considering North, Center, and South of Italy (data not shown).

**Table 2:** Smoking and sleep habits before and during COVID-19 emergency

	Smoking pre-COVID-19	Smoking during COVID-19
No	2646 (74.9)	2762 (78.2)
< 5 cigarettes/day	313 (8.9)	289 (8.2)
5–10 cigarettes/day	295 (8.3)	223 (6.3)
> 10 cigarettes/day	279 (7.9)	259 (7.3)

	Sleep habits pre-COVID-19	Sleep habits during COVID-19
< 7 h/night	1722 (48.7)	1275 (36.1)
7–9 h/night	1763 (49.9)	1935 (54.8)
> 9 h/night	48 (1.4)	323 (9.1)

Values are expressed as number and percentage (n(%))

**Fig. 1:**
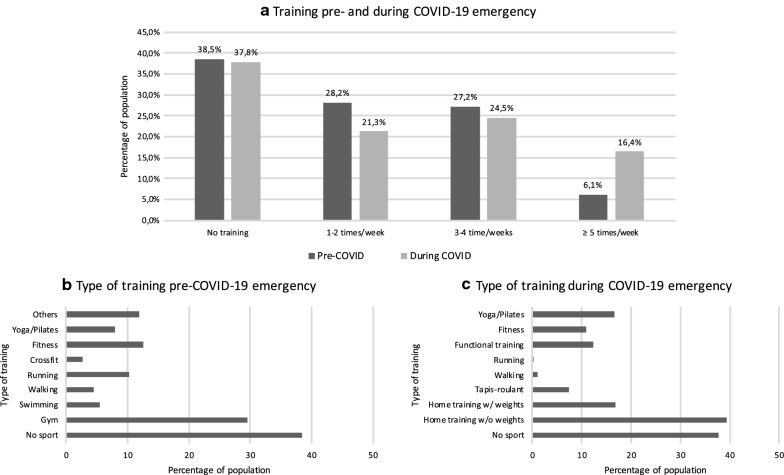
Frequency (**a**) and type of training (**b**, **c**) before and during the COVID-19 emergency

Concerning physical activity, no significant difference between the percentage of people that did not train before (37.7%) nor during (37.4%) the COVID-19 lockdown was observed (p = 0.430). On the contrary, a higher frequency of training during the emergency was found when compared to the previous period (McNemar value = 259.529, p < 0.001). Data about frequency and type of training are reported in Fig. 1.

### Eating habits changes during COVID-19 emergency

With regards to eating habits, more than half of the participants feel a change in their hunger/satiety perception: 627 (17.7%) and 1214 (34.4%) of them have less or more appetite, respectively. The multinomial logistic regression showed that changed work habits (suspension or smart working), in comparison to unchanged ones, and female gender are associated to modified appetite, either negatively and positively (job: OR = 1.791, p < 0.001; OR = 1.431, p < 0.001; sex: OR = 1.521, p < 0.001; OR = 1.738, p < 0.001). Moreover, North and Center Italy are both inversely associated to appetite increase when compared to the South and Islands (OR = 0.527, p < 0.001; OR = 0.582, p < 0.001). The Kruskal–Wallis analysis and the post hoc test have also shown a significant difference in age among the three groups (p < 0.001). People who declare an increased appetite are younger than those with unchanged or reduced one. No difference was found for BMI. Notably, 1199 (33.9%) subjects declare to feel hungry before the main meals, 807 (22.8%) in between them and 395 (11.2%) after dinner. As expected, the binary logistic regression showed after-dinner hungry to be associated to the habit of having a break before bedtime (OR = 4.067, p < 0.001). Moreover, BMI and age were found to be positively and inversely associated to the increased appetite and night snacks, respectively (OR = 1.073, p < 0.001; OR = 0.972, p < 0.001). Living in Center and Southern Italy and Islands resulted to be associated to the after dinner snack in comparison to the Northern region (OR = 1.843, p = 0.009; OR = 2.128, p = 0.002). No difference was found for gender. Interestingly, more than half of the subjects has not changed the number of their daily meals (57.8%), while 17.5% and 23.5% declares to skip or introduce a break or a main meal, respectively.

**Fig. 2:**
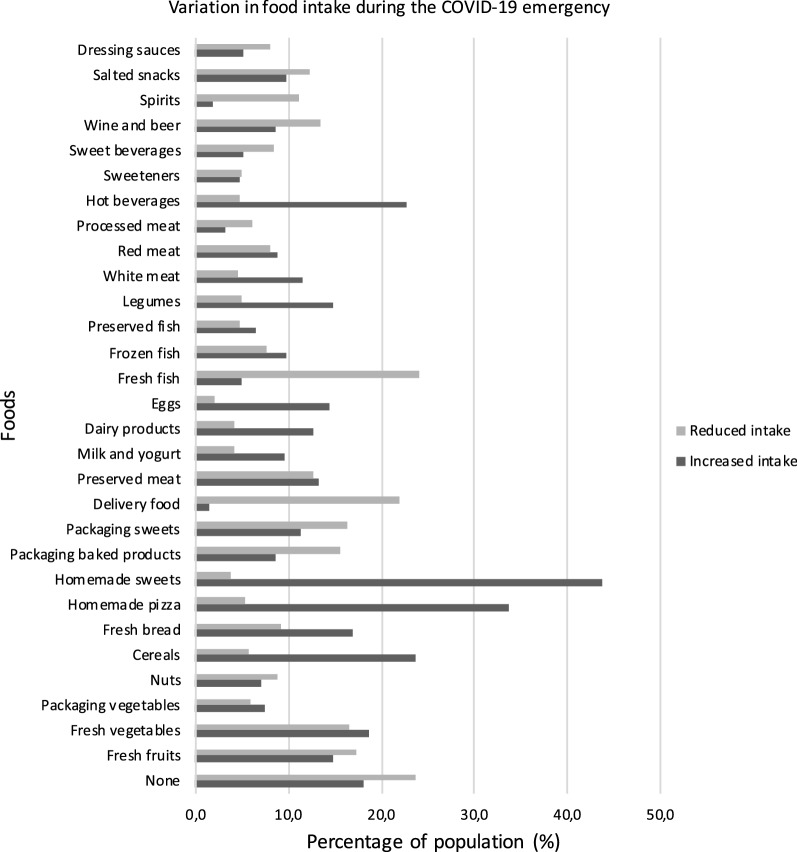
Variation in food intake during the COVID-19 emergency

The survey investigated the variation in food intake during the COVID-19 emergency (Fig. 2). Data show an increase of homemade recipes (e.g. sweets, pizza and bread), cereals, legumes, white meat and hot beverages consumption, and a decrease of fresh fish, packaging sweets and baked products, delivery food and alcoholics intake.

During the COVID-19 lockdown, 37.4% and 35.8% of the study population declares to eat more or less healthy food (fruit, vegetables, nuts and legumes), respectively. No difference between the two groups was found. People who decrease the junk food consumption (29.8%) were significantly more representative than those who increase it (25.6%) (r^2^ = 9.560, p = 0.002). Binary logistic regression analysis showed that an higher BMI, as well as a lower age, were associated to an increase of junk food consumption (packaged sweets and baked products, sweet beverages, savory snacks and dressing sauces) (OR = 1.025, p = 0.005; OR = 0.979, p < 0.001). An enhanced appetite and after dinner hunger were both associated with an increased risk of junk food intake (OR = 4.044, p < 0.001; OR = 1.558, p < 0.001). In the multivariable model, the association remains significant for all the variables, except for after dinner hunger. On the contrary, no association was found between BMI, age and the increase of healthy food intake (p = 0.381, p = 0.053). Moreover, a reduced appetite was related to a major consumption of healthy foods (OR = 1.718, p < 0.001). The perception of weight gain has been detected to be positively and inversely associated to the increase consumption of junk food or healthy food, respectively (OR = 3.122, p < 0.001; OR = 0.805, p = 0.002), to a higher BMI (OR = 1.073, p < 0.001) and to the female gender (OR = 1.234, p = 0.008). Moreover, people who have suspended their usual job or started smart working have a greater perception of having increased their weight when compared to subjects who did not change their job routine (OR = 1.250, p = 0.037). Finally, people who declare to train during the lockdown, as well as people from North and Center of Italy in comparison to those from the Southern and Islands, resulted to have a minor perception of weight gain (OR = 0.660, p < 0.001; OR = 786, p = 0.024; OR = 0.747, p < 0.001). No association was found with the age of the population (p = 0.340).

Most of the population purchases food at the supermarket (75.8%), 26.0% at the grocery shops, 14.8% at farmers, organic or local markets or using Solidal Purchasing Groups, and 9.0% uses online delivery. Finally, 11.8% of participants declare not to purchase food and to delegate shopping to third parties. More than half of the population (54.0%) declares to use the leftover food more than 30% of times. The binary logistic regression showed that shopping at farmers, organic markets, local markets or using Solidal Purchasing Groups was associated to the habit of recycling the leftover food (OR = 1.468, p < 0.001). Moreover, people from the North and Center of Italy appeared to be more prone to this behavior when compared to Southern and Islands population (OR = 2.109, p < 0.001; OR = 1.735, p < 0.001). No association was found with age and gender.

### Adherence to the MD

To assess the compliance to the MD recommendations during the COVID-19 lockdown, the MEDAS questionnaire was included in the survey.

After participants stratification in 3 classes on the basis of the MEDAS values, differences in the compliance rates for each food were calculated and depicted in radar charts which illustrate the gap between the current state (percentage of participants currently adherent to each dietary recommendation) and the ideal situation (100% compliance) (Fig. 2). As expected, among the three classes of adherence to the MD, there were significant differences for most of the items. In particular, in the highest adherence to the MD, the intake of fruit, vegetables, nuts, legumes and fish was respectively: 58.7%, 93.7%, 75.9%, 80.9% and 63.3% (Fig. 2), underlining the improvement of the consumption of typical components of the dietary pattern in our Mediterranean population. Moreover, the consumption of foods not included in the MD profile seems to be reduced.

**Fig. 3:**
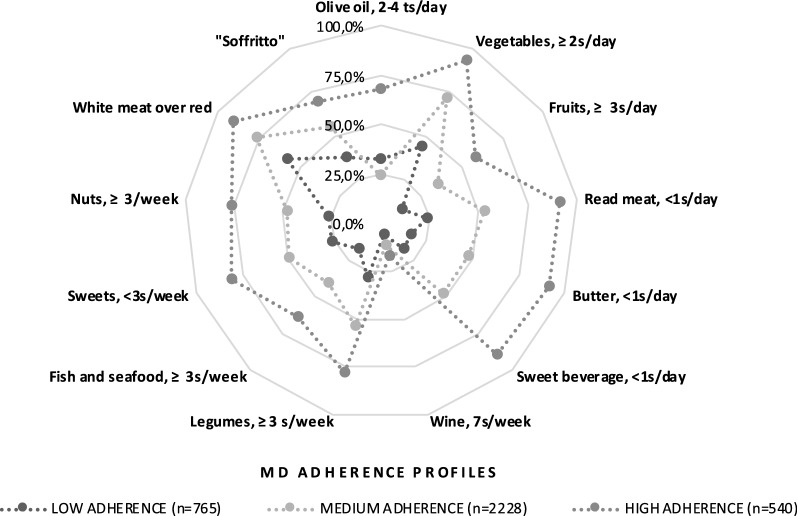
Compliance with items from MEDAS according to high, medium and low adherence to the Mediterranean diet (MD). The radar chart plots the values of each item of MEDAS score along a separate axis that starts in the centre of the chart (0% compliance) and ends at the outer ring (100% compliance). The values are the percentage of the population adherent to each recommendation

**Fig. 4:**
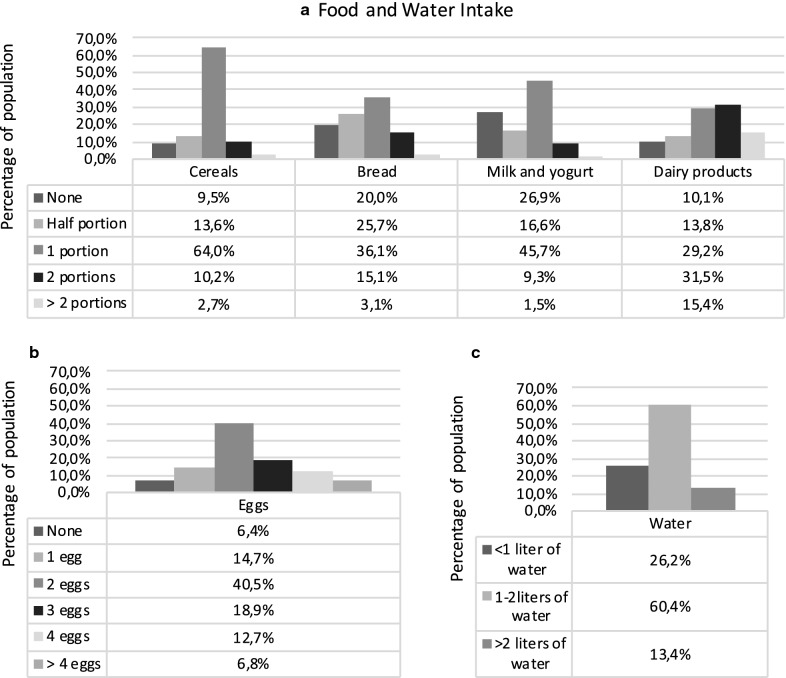
Food and water intake during COVID-19 lockdown. **a** None, half, 1, 2 and > 2 represent the number of daily servings of cereals, bread, milk and yogurt and dairy products. Pasta, rice or other cereals (spelled, barley, oats, quinoa) daily serving: 1 medium serving = 80 g. Bread daily serving: 1 medium serving = 80 g or 2 slices. Milk or yogurt daily serving: 1 serving = 150 ml in a cup or 125 g a jar. Cheese or dairy products weekly serving: 1 portion of dairy product = 100 g; 1 portion of matured cheese = 50 g. **b** None, 1, 2, 3, 4 and > 4 represent the number of weekly servings of eggs. **c** < 1 L, 1–2 L and > 2 L represent the daily intake of water. The ordinate axis represents the percentage of population. The abscissa axis represents the daily/weekly portions for each category of foods

In Table 3, the results of positive answers to MEDAS questionnaire and the adherence to MD are reported. The Kruskal–Wallis test showed a significant difference of MEDAS score among the three Italian areas (p = 0.004), with significant higher scores in Northern and Southern Italy and Islands when compared to Center Italy (post hoc analysis p = 0.011, p = 0.048). Moreover, an inverse correlation was found between MEDAS score, BMI and age (r = 0.096, p < 0.001; r = 0.066, p < 0.001). In particular, the population group aged 18-30 years resulted to have a higher MEDAS score when compared to the younger and the elder population (p < 0.001; p < 0.001, respectively). Moreover, normal-weight people have a significant greater level of adherence to MD in comparison to overweight and obese ones (p < 0.001; p < 0.001). No difference was found among the other classes of BMI. Finally, no difference between gender and employment status groups was found for the MEDAS score. Furthermore, participants were asked to indicate how many daily/weekly servings of food groups not included in MEDAS they consumed. Results are shown in Figs. 3 and  4.

**Table 3:** Positive answers to MEDAS questionnaire and adherence to the MD

	Whole sample (n = 3533)	Northern Italy (n = 547)	Center Italy (n = 2009)	Southern Italy and Islands (n = 977)
Olive oil, main dressing	*3368 (95.8)*	*518 (94.7)*	*1940 (96.6)*	*928 (95.0)*
Olive oil, >= 4 ts/day	*1827 (51.7)*	257 (47.0)	*1076 (53.6)*	*494 (50.6)*
Vegetables, >= 2 s/day	*2430 (68.8)*	*398 (72.8)*	*1396 (69.5)*	*636 (65.1)*
Fruits, >= 3 s/day	1202 (34.0)	180 (32.9)	666 (33.2)	356 (36.4)
Read meat, < 1 s/day	*1854 (52.5)*	*307 (56.1)*	*1039 (51.7)*	*508 (52.0)*
Butter, < 1 s/day	1668 (47.2)	301 (55.0)	888 (44.2)	479 (49.0)
Sweet beverage, < 1 s/day	1676 (47.4)	293 (53.6)	916 (45.6)	467 (47.8)
Wine, 7 s/week	396 (11.2)	60 (11.0)	245 (12.2)	91 (9.3)
Legumes, >=3 s/week	*1826 (51.7)*	267 (48.8)	966 (48.1)	*593 (60.7)*
Fish and seafood, >= 3 s/week	1376 (38.9)	198 (36.2)	750 (37.3)	428 (43.8)
Sweets, < 3 s/week	1753 (49.6)	*280 (51.2)*	970 (48.3)	*503 (51.5)*
Nuts, >= 3/week	1675 (47.4)	281 (51.4)	909 (45.2)	485 (49.6)
White meat over red	*2653 (75.1)*	*427 (78.1)*	*1515 (75.4)*	*711 (72.8)*
“Soffritto”	*1890 (53.5)*	*309 (56.5)*	*1067 (53.1)*	*514 (52.6)*
MEDAS score	7 [6–9]*	7 [6–9]	7 [6–9]	7 [6–9]
Adherence to the MD				
Low	765 (21.7)	108 (19.7)	463 (23.0)	194 (19.9)
Medium	2228 (63.1)	344 (62.9)	1261 (62.8)	623 (63.8)
High	540 (15.3)	95 (17.4)	285 (14.2)	160 (16.4)

Positive answers to MEDAS questionnaire. Compliance rates of at least 50% are indicated in italics. Data are expressed as number and percentage in parenthesis (n (%)) for categorical variables or median and IQR in square brackets (M [IQR]) for continuous variables. Vegetables daily serving: 1 medium portion = 200 g. Fruit daily serving: 1 serving = 100–150 g portion. Red meat/hamburgers/other meat daily serving: 1 medium portion = 100–150 g. Butter, margarine or cream daily serving: 1 medium portion = 12 g. Sweet or sugar sweetened carbonated beverages daily serving: 1 medium portion = 200 ml. Wine daily serving: 1 medium portion = 125 ml. Legumes weekly serving: 1 portion = 150 g. Fish daily serving: 1 medium portion = 100–150 g. Seafood daily serving: 1 medium portion = 200 g. Nuts weekly serving: 1 portion of dairy product = 30 g

*MEDAS* Mediterranean diet adherence screener; *MD* Mediterranean diet; *s* serving; *ts* tablespoon

*The Shapiro–Wilk test was performed to evaluate variables distribution. Variables are considered non-normally distributed for p < 0.05

## Discussion

This population-based study provides a snapshot of the eating habits and lifestyle of Italian residents, who participated in the survey between 5th and 24th of April 2020, after 7 weeks of lockdown.

To our knowledge, this study was among one of the first to investigate the immediate impact of the COVID-19 lockdown on eating habits and lifestyle changes among Italian residents. The web-survey was concluded on 24th of April 2020 as it was the first day in Italy with the same number of newly infected and cured people. To that date, according to National Civil Protection Service data [36], the total number of assessed cases in Italy was 192,994: 106,527 people have tested positive; 60,498 patients have recovered; 25,969 died as confirmed only upon certification of cause of death by the Istituto Superiore di Sanità (ISS). In detail, there were in the North of Italy: 34,368 positive cases in Lombardy, 12,509 in Emilia-Romagna, 15,391 in Piedmont, 9679 in Veneto, 2920 in Trentino Alto Adige, 1320 in Friuli Venezia Giulia, 3437 in Liguria, 354 in the autonomous province of Aosta Valley. In Central Italy: 6133 in Tuscany, 4492 in Lazio, 3273 in Marche, 322 in Umbria. In South Italy: 2943 in Campania, 2933 in Apulia, 2079 in Abruzzo, 821 in Calabria, 229 in Basilicata and 200 in Molise. In Islands: 2320 in Sicily and 804 in Sardinia (Additional file 1: Figure S1).

Therefore, we decided to analyse the eligible data by dividing the population according to the regions of Northern Italy (Piedmont, Aosta Valley, Lombardy, Liguria, Emilia-Romagna, Veneto, Friuli Venezia Giulia, Trentino Alto Adige), the Center (Tuscany, Lazio, Marche, Umbria, Abruzzo, Molise and San Marino Republic) the South (Campania, Basilicata, Calabria, Apulia) and the Islands (Sicily and Sardinia), to evaluate the weight of the responses based on the singular emotional state of each individual, but also based on the severity of the epidemic and the number of sick and dead cases. Territorial coverage of our web survey extends to all Italian Regions, and ranges from a minimum of 15.48% (Northern Italy) to a maximum of 56.86% (Center Italy). Female respondents are about triple compared to male respondents. The strategy adopted by many countries, including Italy, to reduce the spread of COVID-19 has been “social distancing”. The lockdown had the positive effect of flattening the epidemic curve, thanks to the maintenance of the social rules imposed. However, the fear of the disease and death, as well as the restrictions of individual freedom, worsened the stress load and produced alteration of habitual behaviors. Accordingly, the lifestyle and eating habits changed during the COVID-19 pandemic period, particularly in 37.3% of respondents, but only 16.7% of them improved their behaviors. A recent review underlines that a balanced nutrition, which can help in maintaining immunity, is essential for prevention and management of viral infections [37]. Considering that COVID-19 has no effective preventive and pharmacological therapies available, healthy eating habits are crucial and elective micronutrient supplementations (e.g. vitamins, trace elements, nutraceuticals and probiotics) may be beneficial especially for vulnerable populations, such as the elderly [37].

During the COVID-19 lockdown, the sense of hunger and satiety changed for more than half of the population: 17.8% of responders had less appetite, while 34.4% of responders increased appetite. The increased sense of hunger and the consequent change in eating habits could justify the perception of weight gain observed in 48.6% of the population. In fact, 40.3% thinks they have slightly increased their weight, while 8.3% of the studied population thinks they have highly increased their weight. On the other hand, 3.3% of smokers in this period have quit smoking. It is interesting to notice that the number of those who smoked more than 10 cigarettes per day has decreased by 0.5%. This phenomenon could be explained by the fear induced in smokers of the increased risk of respiratory distress and mortality from COVID-19 [38]. Those who did not use to play sports before the COVID-19 lockdown did not use this as an opportunity to start. However, the most interesting fact is that among those who already took part in sports, training frequency has increased. Those who previously managed to exercise only occasionally, now have more time to do it at home. The percentage of those who train five or more days a week has gone from 6 to 16%, with an average increase of 9.9%. A slight increased physical activity has been reported, especially for bodyweight training (38.3% of respondents).

Interestingly, more than half of the subjects have not changed the number of their daily meals (57.8%), while 17.5% and 23.5% declare to skip or introduce a break or a main meal, respectively.

15% of those interviewed turned to farmers or organic purchasing groups for fruit and vegetables, whose consumption did not decrease despite the enormous difficulties of the agricultural supply chain. During the lockdown, Italians have more desire to cook, and above all to knead. Accordingly, the consumption of homemade desserts, bread and pizza has increased. On the other hand, the consumption of savory snacks, snacks, processed meat, carbonated and sugary drinks has decreased.

It was expected that during the quarantine there would have been a reduction of the consumption of fresh food, accompanied by vitamins and minerals deficiency, including vitamin C and vitamin E and beta-carotene with antioxidants and anti-inflammatory properties. The deficiency of these micronutrients is associated with both obesity and impaired immune responses, thus making more susceptible to viral infections [39, 40]. However, during the lockdown, Italians have paid attention to Mediterranean food, and the nutritional quality has remained high, especially in Northern and Central Italy, areas in which there is also a lower BMI compared to the areas of Southern Italy and the Islands (p < 0.05) [41]. We suggest that MD could represent one of the best food models to restore innate and adaptive immunity and might be an adjuvant therapeutic choice of COVID-19.

Obesity is a state of chronic low-grade inflammation dependent on the adipokine secretion of the adipose tissue with immunomodulatory effects [42] that contributes to the onset of several metabolic diseases (including insulin resistance and type 2 diabetes mellitus, dyslipidemia and hypertension). These, due to the downregulation of the innate and adaptive immune responses, make the immune system more vulnerable to infections, resulting in patients being less responsive to vaccinations, antivirals and antimicrobial drugs [43]. These immunomodulatory effects may contribute to aggravate respiratory viral infections [11]. Thus, even if to date there is no evident data reporting that individuals with obesity have a higher risk of getting COVID-19, it is known that more severe forms of respiratory failure are present in patients with obesity. Therefore, it could be hypothesized that individuals with obesity could be at higher risk of serious illness if infected.

The survey explored the perception of body weight changes: 37.4% of the study population declares a stable weight, 13.9% believes to have lost weight, 40.3% feels to have a slight weight gain, and 8.3% to have gained a lot of weight. The perception of weight gain resulted to be present in people who started the smart working, especially in the North and Center of Italy, the same zone in which an increase of physical activity was observed. Therefore, it is strongly recommended to reduce the consumption of junk food to decrease “obesogenic environment” which predisposes to weight gain and susceptibility to COVID-19 [44, 45].

In the present study, we provided for the first time data on the Italian population adherent to the MD pattern during the COVID-19 lockdown, observing that there has not been a deterioration. According to our previous data obtained from a survey, that was conducted to identify clusters of eating patterns among the Italian population aged 15–64 years, three clusters were identified: “Mediterranean-like”, “Western-like” and “low fruit/vegetables”. Among the 5278 subjects, the “Mediterranean-like” pattern was more common among females and elderly; the other clusters were significantly associated with obesity [46]. Indeed, with great surprise, we realized that the most careful in eating Mediterranean food were the young people of the 18–30 age group (p < 0.001; p < 0.001, respectively). Results from the MEDAS questionnaire in our population sample, classified according to the degree of adherence to the MD demonstrated that subjects with low, medium and high adherence to the MD, had adequate consumption, more than 50% of some typical MD food such as olive oil (94.7% in Northern Italy, 96.6% in the Center Italy and 95% in Southern Italy and Island), vegetables (68.8% in Northern Italy, 72.2% in the Center Italy and 69.5% in Southern Italy and Island), legumes (51.7% in Northern Italy, and 60.7% in Southern Italy and Island). In all the three zones, there is an adequate use of slightly fried, known as “soffritto”. Nutritional status is an important form of protection against the emergence of new viral pathogens [47]. Therefore, a correct diet rich in nutrients with antioxidant and anti-inflammatory activities, such as that suitable for MD, helps to reduce virulence of SARS-Cov-2 [48, 49].

An inadequate intake of Mediterranean foods exposes the whole population to specific oxidative damage [28], and, thus, to susceptibility to COVID-19. Our results comfort that the inflammation and oxidative damage, dependent on the consumption of junk and ultra-processed food, in the postprandial period contribute significantly to a greater susceptibility to develop chronic diseases that cannot be communicated, whereas the consumption of seasonal foods and foods rich in antioxidants is highly protective [50].

The main limitation of the present study is represented by a self-reported questionnaire, which may lead to the actual misreporting of data. However, our web-survey was similar to others that have been frequently employed. A strength of our study was represented by the fact that the survey was conducted quickly in the most critical period of the epidemic in Italy, less than three weeks after the lockdown.

## Conclusions

In this study, we have provided for the first time data on the Italian population lifestyle, eating habits and adherence to the Mediterranean diet pattern during the COVID-19 lockdown. The perception of weight gain was observed in 48.6% of the population, whereas a slight increased physical activity has been reported in 38.3% of respondents, especially for bodyweight training. Interestingly, the population group aged 18–30 years resulted to have a higher adherence to the MD when compared to the younger and the elder population. Moreover, 15% of respondents turned to farmers or organic purchasing groups for fruit and vegetables, especially in the North and Center of Italy, where BMI values were lower. Another positive result is the percentage reduction in smokers by 3%. However, as the COVID-19 pandemic is still ongoing, our data need to be confirmed and investigated in future larger population studies.

### Supplementary information

**Additional file 1. Figure S1.** Geographical distribution of COVID-19 total positive cases in Italy on April 24th 2020. Data derived from the Health Ministry of Italy [51].

## Data Availability

All data generated or analysed during this study are included in this published article.

## Appendix: Questionnaire

**Table:**

Questions	Answers
*Personal data*	
1. Age	Age in number
2. Gender	Female/male
3. Hometown	Province
4. Current employment	Unemployed/ Retiree/ Student/ I work in smart working at home/ I go to the work as usual/ I have currently suspended my job
*Anthropometrics*	
5. Weight	Weight in kg
6. Height	Height in cm
*MEDAS*	
7. Is olive oil the main culinary fat used?	Yes/No
8. Are ≥ 4 tablespoons of olive oil used each day?	Yes/No
9. Are ≥ 2 servings (of 200 g each) of vegetables eaten each day?	Yes/No
10. Are ≥ 3 servings of fruit (of 80 g each) eaten each day?	Yes/No
11. Is < 1 serving (100-150 g) of red meat/hamburgers/other meat products eaten each day?	Yes/No
12. Is < 1 serving (12 g) of butter, margarine or cream eaten each day?	Yes/No
13. Is < 1 serving (330 ml) of sweet or sugar sweetened carbonated beverages consumed each day?	Yes/No
14. Are ≥ 3 glasses (of 125 ml) of wine consumed each week?	Yes/No
15. Are ≥ 3 servings (of 150 g) of legumes consumed each week?	Yes/No
16. Are ≥ 3 servings of fish (100-150 g) or seafood (200 g) eaten each week?	Yes/No
17. Is < 3 servings of commercial sweets/pastries eaten each week?	Yes/No
18. Is ≥ 1 serving (of 30 g) of nuts consumed each week?	Yes/No
19. Is chicken, turkey or rabbit routinely eaten instead of veal, pork, hamburger or sausage?	Yes/No
20. Are pasta, vegetable or rice dishes flavoured with garlic, tomato, leek or onion eaten ≥ twice a week?	Yes/No
Dietary habit	
21. How many portions of pasta, rice or other cereals (spelled, barley, oats, quinoa) do you consume per day? (1 medium portion = 80 g)	None/Half portions/1 portion/2 portions/> 2 portions
22. How many portions of bread do you consume per day? (1 medium portion = 80 g or 2 slices)	None/Half portions/1 portion/2 portions/> 2 portions
23. How many portions of milk or yogurt do you consume per day? (1 serving = 150 ml in a cup or 125 g a jar)	None/Half portions/1 portion/2 portions/> 2 portions
24. How many portions of cheese or dairy products do you consume per week? (1 portion of dairy product = 100 g; 1 portion of matured cheese = 50 g)	None/Half portions/1 portion/2 portions/> 2 portions
25. How many eggs do you consume per week?	None/1 egg/2 eggs/4 eggs/> 4 eggs
26. Did your lifestyle and eating habits changed during the COVID-19 pandemic period ?	No, they didn’t/yes, it get worse/yes, it improved
27. During this period, which of these foods are you consuming MORE than before?	None/fruits/fresh vegetables/frozen vegetables/nuts/pasta and cereals/bread/homemade pizza/homemade pastries/industrial bakery products/sweets/ham and processed meat/dairy products/cheese/cow’s milk and yogurt/vegetable drinks/eggs/fish/frozen fish/canned fish/legumes/white meat/red meat/coffee, tea, herb tea/sugar or sweeteners/sugary and sparkling drinks/wine, beer/alcoholic drinks/snacks/seasoning sauces/other
28. During this period, which of these foods are you consuming LESS than before?	None/fruits/fresh vegetables/frozen vegetables/nuts/pasta and cereals/bread/homemade pizza/homemade pastries/industrial bakery products/sweets/processed meat/dairy products/cheese/cow’s milk and yogurt/vegetable drinks/eggs/fish/frozen fish/canned fish/legumes/white meat/red meat/coffee, tea, herb tea/sugar or sweeteners/sugary and sparkling drinks/wine, beer/alcoholic drinks/snacks/seasoning sauces/other
29. Did you change the number of daily meals, during this period?	No, it did’t/Yes, I skip 1 or more of the main meals (breakfast, lunch, dinner)/Yes, I skip 1 or more of snacks between meals/Yes I added 1 or more of the main meals/Yes, I added 1 or more of the snacks between meals/Yes, I eat out of the meals
30. How much water do you drink per day?	< 1 L/1 L–2 L/> 2 L
31. Do you eat the leftover food?	Never/< 10%/10–30%/30–50%/> 50% of the time
*Lifestyle Habits Changes*	
32. Where do you buy your food and essentials during this period?	I do not go out for shopping/supermarket/grocery store/Local street market/farmer’s market/organic food shop/fairtrade market/other
33. Did you smoke before COVID-19 pandemic period? (cigarettes, cigarillos, cigars, electronic cigarette)	No/Yes, < 5 cigarettes/Yes, 5–10 cigarettes/Yes, > 10 cigarettes
34. Do you currently smoke?	No/Yes, < 5 cigarettes/Yes, 5–10 cigarettes/Yes, > 10 cigarettes
35. How many hours did you sleep before the COVID-19?	<7 h per night/7–9 h per night/> 9 h per night
36. How many hours do you currently sleep?	<7 h per night/7–9 h per night/> 9 h per night
37. Did you play sport before the COVID-19 emergency?	No/gym/run/swimming/soccer/volleyball/basket/crossfit/dance/yoga/aerobic fitness/martial arts/tennis/aerial gymnastics/other
38. How many times did you play sport?	I didn’t practice any sport/1–2 times a week/3–4 times a week/> 5 times a week
39. Are you currently playing sport at home?	No/weightless workout/weight training at home/tapis roulant/functional training/yoga/postural gymnastics/other
40. How many times do you play sport at home?	I don’t practice any sport/1–2 times a week/3–4 times a week/> 5 times a week
41. What is the time of the day when you are particularly hungry?	Before main meals/between main meals/After dinner
42. Did your sense of hunger and satiety change during the period at home for the COVID19 emergency?	No/Yes, less appetite/Yes, more appetite
43. Did you gain weight during the COVID-19?	No, my weight is stable; No, I think I lose weight/Yes, I think I gain not so much weight/Yes, I think I gain a lot of weight

## Abbreviations

BMI: Body mass index
MD: Mediterranean diet
MUFA: Monounsaturated fatty acid
EHLC-COVID19: Eating habits and lifestyle changes in COVID19 lockdown
MEDAS: Mediterranean diet adherence screener
ISS: Istituto Superiore di Sanità
