# Supplementary figures and images for: Eating habits and lifestyle changes during COVID-19 lockdown: an Italian survey
Source: J Transl Med. 2020 Jun 8;18:229. doi: 10.1186/s12967-020-02399-5 (PMC7278251; doi:10.1186/s12967-020-02399-5)

# COVID-19 Total positive cases on April 24<sup>th</sup> 2020

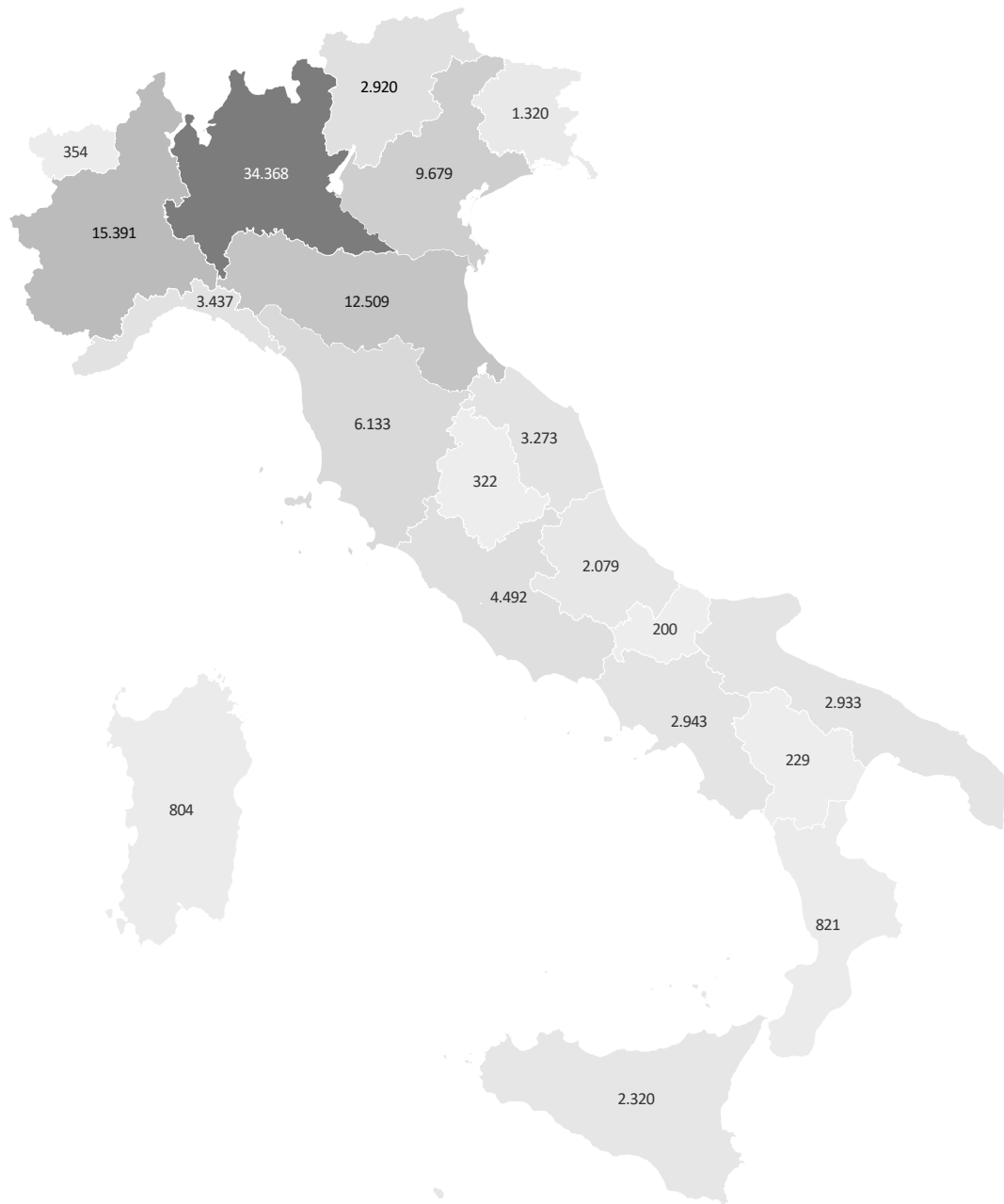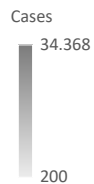

Supplement: Supplementary file 1 — Additional file 1. Figure S1. Geographical distribution of COVID-19 total positive cases in Italy on April 24th 2020. Data derived from the Health Ministry of Italy [51]. [file 12967_2020_2399_MOESM1_ESM.pdf]
